# The effectiveness of non-pharmacological healthcare interventions for asthma management during pregnancy: a systematic review

**DOI:** 10.1186/1471-2466-14-46

**Published:** 2014-03-19

**Authors:** Elida Zairina, Kay Stewart, Michael J Abramson, Johnson George

**Affiliations:** 1 Centre for Medicine Use and Safety, Monash University, Parkville, VIC, Australia; 2 Department of Epidemiology and Preventive Medicine, Monash University, Melbourne, VIC, Australia; 3 Department of Allergy, Immunology and Respiratory Medicine, The Alfred Hospital, Melbourne, VIC, Australia

**Keywords:** Asthma, Non-pharmacological, Interventions, Management, Pregnancy

## Abstract

**Background:**

While reviews have been published on asthma management in pregnant women, none has examined the effectiveness of non-pharmacological healthcare interventions for optimizing asthma management in pregnant women. This systematic review aims to identify non-pharmacological healthcare interventions for optimizing asthma management during pregnancy and to examine their effects on maternal asthma control and neonatal outcomes.

**Methods:**

The Cochrane Central Register of Controlled Trials (CENTRAL, Cochrane Library), MEDLINE, EMBASE, PsycINFO, CINAHL Plus and International Pharmaceutical Abstracts (IPA) were searched. Two reviewers independently assessed the identified studies against the eligibility criteria and extracted relevant information. The effects of the intervention were assessed qualitatively.

**Results:**

Nine studies were identified, of which six were rejected according to the exclusion criteria. The three studies included in the final review described an education program, progressive muscle relaxation (PMR) and Fraction of exhaled Nitric Oxide (FeNO) guided management of asthma in pregnant women. The PMR and FeNO-guided interventions showed significant improvements in maternal asthma control (lung function and quality of life) and neonatal outcomes (birth weight).

**Conclusions:**

Further evidence from well-designed studies evaluating non-pharmacological healthcare interventions for optimizing asthma management in pregnant women is required.

## Background

Asthma is one of the most serious health problems affecting people of all ages throughout the world [1,2]. In the United States of America the prevalence of self-reported asthma among pregnant women was between 8.4% and 8.8% during the period 1997 to 2001, and 4.1% of all pregnant women had experienced an asthma attack in the previous year [3,4]. In Australia, asthma is the most common chronic disease affecting pregnant women, complicating one in eight pregnancies [5].

A prospective study conducted by Schatz et al. [6] of 366 pregnancies in 330 women with asthma, showed that during pregnancy, asthma improved in slightly more than a quarter of patients (28%), worsened in slightly more than a third of patients (35%) and remained unchanged in a third of patients (33%). More than half of women with asthma do not take their asthma preventer medications on a regular basis before and during pregnancy, leading to asthma exacerbations [3,7]. Good asthma control during pregnancy is important to reduce risks for both mother (e. g. pre-eclampsia, perinatal mortality, and need for cesarean delivery) and infant (e.g. low birth weight and prematurity) [8,9]. Therefore, pregnant women with asthma warrant additional support comprising education, ongoing monitoring and review of treatment [10].

Many national and international bodies have developed guidelines for asthma management in pregnancy. They include the British Thoracic Society, National Heart Lung and Blood Institute (NHLBI), American College of Obstetricians & Gynecologists (ACOG) and the American College of Asthma and Allergy (ACAAI), National Asthma Council of Australia (NAC), and Global Initiative for Asthma (GINA) [11-15]. All these guidelines have emphasized the need to provide optimal therapy to maintain control of asthma throughout gestation for maternal health and quality of life as well as for normal fetal maturation. The Expert Panel Report of the Working Group on Asthma and Pregnancy – Updates in National Asthma Education and Prevention Program (NAEPP) – has recommended four critical components for managing asthma during pregnancy: (1) assessment and monitoring of asthma including objective measures of pulmonary function, (2) control of factors contributing to asthma severity, (3) patient education, and (4) a stepwise approach to pharmacological therapy [16]. Asthma management during pregnancy requires close collaboration among obstetricians, primary care physicians, and asthma-care specialists [17]. Better asthma control can be achieved if patients are involved in self-management, including self-monitoring of either symptoms or peak expiratory flow rates, maintaining regular contact with medical practitioners and following written asthma action plans [18].

While there are many published reviews of pharmacological asthma management in pregnant women [19,20], none has assessed the effectiveness of non-pharmacological healthcare interventions for optimizing asthma management in pregnant women. Most of the interventions in pregnant women have focused on the safety and efficacy of asthma medications in pregnant women [21,22]. General practitioners (family physicians) have reported a lack of confidence and/or knowledge in managing deteriorating asthma in pregnancy, although having a good understanding of the safety of asthma medications during pregnancy [23]. Despite being concerned about health outcomes, women are not well supported in managing asthma during pregnancy [24]. Empirical evidence on interventions to optimize asthma management during pregnancy, targeting both pregnant women with asthma and their health professionals, is needed. The aim of this review was to identify non-pharmacological healthcare interventions for optimizing asthma management during pregnancy and examine their effects on maternal asthma symptoms and neonatal outcomes.

## Methods

### Eligibility criteria

To be included, studies had to describe the effectiveness of non-pharmacological healthcare interventions for managing asthma in pregnant women using one of the following prospective study designs: randomized controlled trials (RCTs) controlled clinical trials (CCTs), or pre- and post- (uncontrolled before and after) studies. Studies of non-pharmacological healthcare interventions in pregnant women, including behavioral or educational interventions targeting patients, patient self-management programs, patient monitoring and follow-up of asthma management were eligible. Studies were excluded if they were not aimed at pregnant women with asthma, comprised only pharmacological interventions in the absence of intervention by a healthcare professional (e. g. drug trials), or only targeted healthcare professionals (e. g. education to improve prescribing). Studies needed to have measured at least one of the following primary or secondary outcomes at baseline and at follow-up:

#### Primary outcomes for the review

1. Asthma symptom scores measured using any validated instrument (e.g. Juniper’s Asthma Control Questionnaire [ACQ] [25]).

2. Health-related Quality of Life (HRQoL) scores measured using any validated instrument (e.g. Asthma Quality of Life Questionnaire-Marks [AQLQ-M] [26]).

3. Asthma -related scheduled or unscheduled healthcare visits to emergency department (ED), general practitioner (GP), or hospitalization.

#### Secondary outcomes

1. Lung function measurements (e.g. Peak expiratory flow rate [PEFR], Forced expiratory volume in one second [FEV_1_], Forced vital capacity [FVC]).

2. Asthma medication adherence (assessed using a valid instrument or objective data).

3. Neonatal outcomes (e.g. birth weight, survival, Appearance, Pulse, Grimace, Activity, Respiration (APGAR) scores, gestational age).

### Information sources

A systematic search of the following databases was carried out using the Cochrane Central Register of Controlled Trials (CENTRAL, The Cochrane Library 2013), Ovid MEDLINE, Ovid EMBASE, Ovid PsycINFO, CINAHL Plus and International Pharmaceutical Abstracts (IPA). In addition to searching these databases, reference lists from previously published review articles were also searched. The final search was carried out in October 2013.

### Search strategies

Professional librarians were consulted in developing the search strategy for each database. No language restrictions were used and searches were not limited to publication years. The broad terms asthma* AND pregnan* as (text word) were used. The following keywords were entered: asthma* OR wheez* OR, bronchoconstrict* OR bronchospas* AND pregnancy* OR pregnant OR maternal* in combination with clinical trials OR randomized controlled trials OR controlled clinical trials. Additional searches using the Medical Subject Headings (MeSH) ‘asthma’ and ‘pregnancy’ were performed in Medline and PubMed.

### Study selection

One author (EZ) ran the search strategy described above. All studies identified were imported into an Endnote® library (version X6, Thomson Reuters). After removal of duplicates, the remaining titles and abstracts were reviewed by EZ to exclude studies that did not meet the inclusion criteria. Full texts of all studies that were considered relevant on the basis of review of title and abstract were retrieved, read and assessed independently by two reviewers (EZ and JG).

### Data collection process and data items

Using an electronic data extraction form [27], one author (EZ) extracted the data from included studies, which were verified by a second author (JG). Any disagreements and uncertainties were identified and resolved in discussion with an adjudicating third author (KS). Given the clinical heterogeneity of the studies included, a qualitative assessment of the effects of the intervention was performed, based on the methodological quality and the study outcomes. The effects of the intervention were described by comparing the difference in outcome measures from baseline to end of the study between the groups. If more than one outcome was reported, priority was given to validated measures [28].

## Results

### Study selection

**Figure 1:**
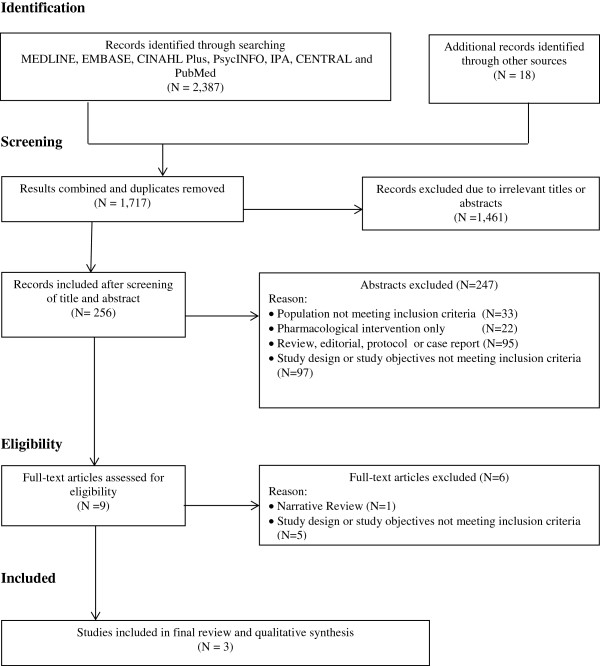
Flow chart of selection process for including studies in the systematic review.

Figure 1 shows the process of selection of studies for the systematic review, based on PRISMA guidelines [29]. Overall, 2,387 references published until 9 October 2013 were identified in the preliminary search: Ovid MEDLINE (n = 636), Ovid EMBASE (n = 779), CINAHL Plus (n = 337), Ovid PsycINFO (n = 22), IPA (n = 137), Ovid CENTRAL (n = 143) and PubMed (n = 333). Screening of the reference lists of published articles resulted in identification of another 18 articles. After combining the results from each database and removal of duplicate titles from Endnote®, 1,717 unique studies remained. After further screening, 1,461 were removed due to irrelevant titles or abstracts, leaving 256 studies for further scrutiny; 247 studies were excluded after further review. Of the nine full-text articles obtained, one was a narrative review [30], one had a retrospective design [31], one had a cross-sectional design [32] and three were based on secondary analysis of other studies [33-35] leaving only three original studies for the final review [36-38].

### Study characteristics

The characteristics and results of the three studies included in the final review are summarized in Table 1. These studies evaluated the following interventions in pregnant women: an education program [36], progressive muscle relaxation (PMR) [37] and management of asthma guided by Fraction of exhaled Nitric Oxide (FeNO) [38]. Heterogeneity in study design, setting, type of intervention, follow-up and outcome measures were found among the three studies. Two studies took place in the antenatal clinic of one Australian hospital [36,38] and another [37] in Germany. All three interventions were conducted in clinics or hospital settings.

**Table 1:** Key features of studies included in the final review

**First Author, Year, study**	**Aim**	**Setting; country**	**Population**	**Study design**	**Interventions**	**Follow-up**	**Outcomes**	**Results**
Murphy, 2005 [36] “Education program”	To determine the level of asthma self-management skills and knowledge, and to implement an asthma education program	Antenatal clinics; NSW, Australia	Pregnant women with a doctor’s diagnosis of asthma (mild, moderate, severe) at ~ 20 weeks gestation	Pre-and post-	I: Received education about asthma control and self-management skills from a nurse (asthma educator) in two visits each consisting of a 30-60 min session (n = 211) C: no control group	~33 week s of gestation (last visit)	Self-reported nonadherence to ICS, lung function (FEV_1_, FEV_1_%, FVC, FEV_1_/FVC), symptoms and reliever medication use.	Non-adherence to ICS decreased (p = 0.006). FEV_1_(*l*) at first visit^1^: mild: 3.14 ± 0.05 moderate: 2.87 ± 0.08 severe: 2.87 ± 0.09 FEV_1_(*l*) at last visit^1^: mild: 3.13 ± 0.08 moderate: 2.91 ± 0.12 severe: 2.95 ± 0.10. No significant difference in lung function, symptoms and reliever medication use between the two visits.
Nickel, 2006 [37] “Progressive Muscle Relaxation (PMR)”	To examine the efficacy of PMR in pregnant women	Psychosomatic clinics; Germany, Austria	Pregnant women with asthma who were regularly seen by an obstetrician/ gynecologist	RCT	I: 30 min PMR session, 3 times a week (n = 32) C: placebo (30 min sham training), 3 times a week (n = 32)	8 weeks from baseline	Lung function (PEF, FEV_1_), QoL (SF-36)	FEV_1_(*l*): initial^2^: I: 1.69 ± 0.6 C: 1.75 ± 0.5 final^2^: I: 2.22 ± 0.5 C: 1.75 ± 0.5 Difference in FEV_1_[95%CI] = 0.5 (0.2 to 0.8) p = 0.005 Difference in SF-36 (mental health component)[95%CI] = 5.8 (1.4 to 10.2) p = 0.01
Powell, 2011 [38] “FeNO based Algorithm”	To test the hypothesis that a management algorithm for asthma in pregnancy based on FeNO and symptoms would reduce asthma exacerbations	Antenatal clinics; NSW, Australia	Non-smoking pregnant women (aged ≥ 18 years) with asthma, 12 – 20 weeks gestation and using asthma medications (e.g. inhaled therapy, beta_2_-agonist) within the past year	Double-blind RCT	I: FeNO algorithm to adjust therapy: (1) FeNO concentration was used to adjust the dose of inhaled corticosteroids (2) ACQ score was used to adjust the dose of long acting beta_2_-agonist (n = 111) C: ACQ- based clinical algorithm (n = 109)	monthly until delivery	Exacerbation types (unscheduled doctor visits, OCS use, hospital admission, ER/labor ward visits), QoL (SF-12 and AQLQ-M), Lung function (FEV_1_ and FEV_1_%), current treatment and perinatal outcomes	Significant reduction in unscheduled doctor visits for asthma (p = 0.002) and OCS use (p = 0.042), QoL (SF-12 mental health component) higher in FeNO group (p = 0.008) – remained significantly different after adjustment for baseline values (p = 0.037), AQLQ-M scores were low at the completion of the study and not different between the groups. FEV_1_(*l*) at randomisation^3^: I: 3.05 (2.96 – 3.14) C: 3.06 (2.96 – 3.15) FEV_1_(*l*) at end of study^3^: I: 3.09 (3.0 – 3.17) C: 3.01 (2.91 – 3.10) No significant difference in lung function.

Data are presented as ^1^) mean ± SE, ^2^) mean ± SD, ^3^) mean [95% Confidence Interval].

ACQ = Asthma Control Questionnaire, AQLQ-M = Asthma Quality of Life Questionnaire-Marks, C = Control, FeNO = Fractional exhaled Nitric Oxide, FEV_1_ = Forced expiratory volume in 1 second, FVC = Forced vital capacity, ICS = Inhaled corticosteroid, I = intervention, min = minute, *l* = liter, OCS = Oral corticosteroid, PEF = Peak expiratory flow rate, QoL = Quality of Life, RCT = Randomized Controlled Trial, SF-36 = Short Form 36, SF-12 = Short Form 12.

### Methodological quality

All three studies had methodological limitations. The education program in the uncontrolled pre- and post-test design study was delivered by the same personnel who were involved in outcome assessment and were not masked (blinded), thus observation/detection bias might have occurred [36]. Only one of the RCTs reported allocation concealment [38]. The outcomes of both trials [37,38] were assessed by masked investigators but only one reported masking of both the participants and personnel who were involved in the intervention [38]. Sample size calculation was described for both RCTs, but not in the pre- and post-test study [36]. Participation and attrition rates varied across the three studies. Only Powell et al. [38] gave reasons for participant withdrawal (11 from the trial group and 6 from the control group). None of the study protocols were published. One trial was registered with the Australian and New Zealand Clinical Trial registry [38].

### Results of individual studies

#### Education program

Murphy et al. [36] conducted a study to implement asthma self-management skills through an education program in pregnant women with asthma in an antenatal clinic. The asthma self-management skills assessed were: medication adherence (inhaled corticosteroid [ICS] users only), knowledge about how the reliever and preventer medications worked and inhaler technique, possession of a written action plan, and self-monitoring. The medication adherence and knowledge were assessed by direct questioning, while the inhaler technique was demonstrated by the patient.

The study found that maternal and neonatal outcomes may be improved by asthma self-management education, which can be delivered in an antenatal clinic by a nurse with specific training in asthma education. Improvements in asthma medication adherence, knowledge and skills were associated with asthma education and should be considered as an important aspect of managing asthma in pregnant women [36]. There were some limitations of this study. The time elapsed (~3 months) between the two visits was identified by the authors as a potential confounder, as changes in asthma control could be influenced by gestation and seasonal changes [36]. Since there was no comparison group, it remained unclear if the asthma management skills improved because of the asthma education provided in the antenatal clinic, as a result of other factors, or spontaneously [36].

#### Progressive Muscle Relaxation (PMR)

This trial by Nickel et al. [37] examined the efficacy of progressive muscle relaxation (PMR) on changes in heart rate, systolic blood pressure (SBP), lung function and quality of life in pregnant women with asthma. The PMR procedures in this study required the participants to monitor and control their state of muscular tension. In the first step, the women deliberately applied tension to certain muscle groups and then released the tension and focused on how the muscle relaxed during the process [37]. This study claimed that the PMR intervention was inexpensive and demonstrated a potential benefit in pregnant women with asthma [37]. Inability to confirm that all the participants followed the instructions was a limitation acknowledged by the authors [37]. The short term follow-up of the study (8 weeks) may have contributed to the low drop-out rate [37]. Only immediate effects were measured after an active intervention. Hence it is unknown if PMR intervention would have had similar effects in the longer term and during asthma exacerbations [37].

#### FeNO-based algorithm

Powell et al. [38] carried out a double-blind parallel group RCT to test whether asthma control in pregnancy would be better using a FeNO-based treatment algorithm compared to an ACQ clinical algorithm in terms of reducing asthma exacerbations. FeNO helps to identify eosinophilic airway inflammation and to adjust the dose of ICS. The FeNO-based algorithm group had a sequential process: (1) FeNO concentration to adjust the dose of ICS; and (2) ACQ score to adjust the dose of long acting beta_2_-agonist. The clinical algorithm was based on asthma control assessed with Juniper’s ACQ questionnaire. The data collected included clinical symptoms, ACQ, quality of life questionnaires (AQLQ-M and SF-12), present treatment (ICS and β_2_ agonist), FeNO and spirometry (FEV_1_, FVC).

This trial showed that the FeNO group had a significantly lower rate of asthma exacerbations during pregnancy (p = 0.001) and unscheduled doctor visits due to asthma during pregnancy (p = 0.002) [38]. However the ACQ scores (symptom-free days) and the AQLQ-M scores of the two groups were not significantly different at the end of the study [38]. The mean daily ICS dose was lower in the FeNO group throughout the study. A higher median birth weight as well as a reduction in preterm deliveries and neonatal hospitalizations was also found in this group [38].

## Discussion

This systematic review evaluated the effectiveness of non-pharmacological healthcare interventions for improving asthma management in pregnant women. The three studies included in the review assessed education, PMR and a FeNO-based algorithm, which were found to have some positive effects on asthma management in pregnancy. Firm conclusions however, cannot be drawn due to the limited number of reported studies, clinical heterogeneity of the interventions, variations in outcome measures and limitations in study designs.

Patient education is the cornerstone of asthma management during pregnancy as it promotes adherence and in turn, improves asthma control [16]. Gibson et al. [39] identified four components of an effective asthma education program: (1) information about asthma and its management, (2) self-monitoring of either symptoms or peak expiratory flow rate, (3) regular medical review for assessing asthma control, severity and medications, and (4) a written action plan to guide patient self-management of asthma exacerbations. Pregnant women with asthma should have a basic understanding of self-monitoring, how to use asthma medications correctly, how to manage worsening asthma and the importance of continued adherence to asthma management plans [16]. Asthma education programs and self-management skills have been proven to be effective in improving health outcomes in adults with asthma [18]. Both the studies of Murphy et al. [36] and Powell et al. [38] provided education to pregnant women on skills and knowledge to manage their asthma, leading to improvement in adherence to medication regimens and asthma action plans.

The 30-min PMR sessions three times a week showed a greater improvement in lung function compared to sham training [37]. A systematic review by Huntley et al. [40] concluded that muscular relaxation may improve lung function. However, there was no evidence for effectiveness on asthma symptoms in pregnant women with asthma. The effects of educational interventions and PMR on asthma at different stages of gestation remain unknown.

The outcomes measured varied among the three studies. Lung function (e.g. FEV_1_, PEF) was measured as an outcome in all of the studies included. All three studies showed some improvement in FEV_1_, although only one study demonstrated a significant improvement in FEV_1_ as a result of the intervention [37]. During pregnancy, static lung function remains the same except for a reduction in functional residual capacity (FRC), expiratory reserve volume (ERV) and residual volume (RV) [41]. As the uterus enlarges, FRC decreases by 10% to 25% of the previous value due to a 35% to 40% decrease in chest wall compliance [42]. Normal pregnancy may have no significant effect on airway function. However in pregnant women with asthma, peak flow rate, FEV_1_ and FVC may decrease particularly during acute exacerbations [43]. The study by Schatz et al. [44] observed that it is important to measure FEV_1_ regularly during pregnancy, both as a prognostic factor for perinatal outcomes and as a measure of asthma control. Monitoring lung function using spirometry is recommended in the initial assessment of all pregnant women with asthma and periodically as needed [45], although lung function is typically impaired only in severe asthma and during acute exacerbations. Further studies are needed to confirm the efficacy of healthcare interventions in pregnant women with asthma over different trimesters, as pulmonary function changes throughout pregnancy in asthmatic women [6,43,46].

Powell et al. [38] reported a significant reduction in the rate of unscheduled doctor visits for asthma and oral corticosteroid use in patients receiving FeNO-guided management. Reduced asthma exacerbations leading to improvements in both maternal and neonatal outcomes were also reported [38]. The application of FeNO as a biomarker of airway inflammation for the adjustments of ICS treatment to guide asthma management has been widely studied, however the results are as yet inconclusive. Several studies have shown that FeNO-guided asthma management is no more effective in reducing asthma exacerbations than current asthma guidelines and conventional pulmonary tests using spirometry [47-50]. Daily FeNO monitoring has been shown to be of no added value compared to daily symptom monitoring. Moreover, FeNO measurements are not routinely available in most clinical settings [11].

The American Thoracic Society [51] recommends FeNO for monitoring airway inflammation in patients with asthma. However, there is insufficient evidence to support more widespread use. Various confounders, including sex, age, height, measurement technique, exhalation flow rate, smoking, anti-inflammatory medications and even what the patient ate for breakfast, may affect FeNO results [51]. A cross-sectional study by Tamasi et al. [32] showed that in pregnant women with asthma, FeNO levels are elevated compared to healthy pregnant women and they correlate with the level of asthma control. Further studies comparing FeNO-guided asthma management with simple implementation of asthma guidelines in conjunction with conventional lung function monitoring, especially in pregnant women, are required to confirm any advantage of FeNO monitoring over traditional monitoring/self-management.

### Strengths and weaknesses of this review

This is the first systematic review of the effectiveness of non-pharmacological healthcare interventions for managing asthma and improving health outcomes in pregnant women with asthma. Unpublished studies were not included in this review. A meta-analysis was not possible because of the clinical heterogeneity of the data and study designs.

### Practice and research implications

A clinical algorithm for asthma management based on objective measures and asthma symptoms could potentially reduce asthma exacerbations during pregnancy. The goals of asthma management in pregnant women are the same as in non-pregnant patients, which are to control asthma symptoms, maximize lung function, minimize medication side effects and prevent asthma exacerbations. These goals need to be considered when designing interventions, in addition to pharmacological treatment, to improve health outcomes in pregnant women with asthma. The cost-effectiveness of interventions and satisfaction of patients and health professionals also need to be assessed before implementation of such interventions in clinical practice. Further evidence is needed from well-designed prospective controlled studies in pregnant women with asthma investigating the effectiveness of interventions that incorporate patient education, patient self-management and periodic follow-up with health professionals.

## Conclusions

Our review suggests that non-pharmacological healthcare interventions including education, self-management, progressive muscle relaxation and periodic follow-up may optimize asthma management in pregnancy. Interventions that enable pregnant women to be monitored regularly using objective measures of lung function or asthma symptoms appear to be more effective in improving health outcomes during pregnancy.

## Abbreviations

ACQ: Asthma control questionnaire; AQLQ-M; AQLQ-M: Asthma quality of life questionnaire – marks; FeNO: Fraction of exhaled nitric oxide; FEV1: Forced expiratory volume in 1 second; FVC: Forced vital capacity; ICS: Inhaled corticosteroid; PEF: Peak expiratory flow rate; PMR: Progressive muscle relaxation; SF- 12: Short form-12; SF-36: Short form-36.

## Competing interests

JG and MA hold an investigator-initiated grant from Pfizer for unrelated research. The other authors declare that they have no conflicts of interest.

## Authors’ contributions

EZ searched the literature and selected the studies. She also extracted and assessed the relevant data, working with JG and KS. EZ drafted the manuscript and developed it with input from all the authors. All authors approved the final manuscript.

## Pre-publication history

The pre-publication history for this paper can be accessed here:

http://www.biomedcentral.com/1471-2466/14/46/prepub
